# Visible After invasion? Ukraine’s representational absence and distortion in global comics before 2014

**DOI:** 10.12688/openreseurope.24247.1

**Published:** 2026-07-24

**Authors:** Svitlana Pidoprygora

**Affiliations:** 1 Department of Slavonic Studies, University of Innsbruck, Innsbruck, 6020, Austria

**Keywords:** comics, Ukraine, representation, popular culture

## Abstract

**Background:**

The representation of Ukraine in global comics has long been shaped by imperial, Soviet, and Russian interpretative frameworks that have obscured Ukrainian historical and cultural specificity. While previous scholarship has examined representations of Eastern Europe in popular culture, comparatively little attention has been paid to the mechanisms through which Ukraine has been rendered visible yet simultaneously displaced within global comics.

**Methods:**

The paper examines three case studies from different comic traditions: *Tintin in the Land of the Soviets* (1929) by Hergé, *Nestor Makhno* (1976) by Spain Rodriguez, and *Ivan Karine* (1963–1964) by Enzo Chiomenti. Combining visual, discursive, and narratological analysis with postcolonial and decolonial perspectives, the article analyses four dimensions of representation: naming, spatial framing, iconography, and narrative positioning. It introduces the concept of distorted visibility to describe representational mechanisms such as absence, substitution, Russification, Sovietization, symbolic appropriation, and mislocation.

**Results:**

The analysis demonstrates that Ukrainian territories, historical figures, and cultural symbols are incorporated into broader Soviet, Russian, or imperial narratives. In Hergé’s work, Soviet space is visually homogenized through Russian cultural markers. Rodriguez presents Nestor Makhno primarily through the framework of the Russian Revolution and anarchist mythology, while Ukraine remains a secondary setting. *Ivan Karine* appropriates Cossack imagery but reorganizes it within a Russian imperial adventure narrative. Ukrainian material becomes visible, while its distinct historical and cultural identity is systematically displaced.

**Conclusions:**

The findings suggest that global comics participated in the production and circulation of enduring cultural assumptions about Ukraine before 2014. The Ukrainian case serves as a lens through which to examine what is made visible – and what remains systematically overlooked – in transnational graphic storytelling. This paper contributes to ongoing discussions on cultural erasure, epistemic asymmetries, and the visual geopolitics shaping representations of Eastern Europe.

## Introduction

The representation of Ukraine in global comics before and after 2014 cannot be separated from broader cultural, historical, and geopolitical processes. Ukraine is not unique in this respect: every country is represented through changing political, cultural, and media frameworks. A particularly persistent structure of representation marks the Ukrainian case. In Western academic, museum, literary, and public discourses, Ukrainian historical figures, cultural phenomena, artistic movements, and geographical spaces have frequently been absorbed into Russian, Soviet, or generically Eastern European frameworks. This is connected not only to external perceptions of Ukraine but also to the historical conditions of Ukrainian statehood: for a long time, Ukraine lacked the political subjectivity and institutional capacity to represent itself internationally as an independent state with its own history, culture, and political agency.

This limited visibility of Ukraine for Western readers has been explicitly acknowledged by scholars and cultural mediators, especially in publications produced after 1991. The collapse of the Soviet Union and Ukraine’s declaration of independence created the conditions for Ukraine to appear more clearly in academic, media, and popular cultural discourses. Yet this visibility remained uneven and often belated. For example, in the German-language volume *Ukraine-Lesebuch: Literarische Streifzüge durch die Ukraine (2006)*, edited by Evelyn Scheer, the afterword notes that many people in Germany still knew little about Ukraine. Ukrainians, Scheer observes, “were rarely perceived as a people in their own right, but were commonly regarded as a regional variant of the Russian nation” [1]. She explains this perception through Ukraine’s long history of alien rule and its post-1945 disappearance behind the *Iron Curtain.* ^1^

This observation is crucial because it shows that Ukraine’s invisibility was not merely a matter of insufficient information. It was also produced by an interpretative habit: Ukrainians were seen through a Russian frame. At the same time, even texts that seek to present Ukraine as a distinct cultural space may still rely on stereotypical visual codes. The cover of *Ukraine-Lesebuch *, with its Cossack figure and allusion to Gogol’s *Taras Bulba*, returns Ukraine to a familiar repertoire of Cossack exoticism, moving within an older symbolic field.

Historian Andreas Kappeler makes a similar argument. He emphasizes that “Ukraine’s sudden appearance in European politics after 1991 came as a surprise to the West. For decades, the large country in the south-west of the Soviet Union had barely been noticed. More than forty million ethnic Ukrainians were usually regarded as a regional subgroup of Russians. Ukrainians and their country were scarcely present in public consciousness, the media, politics, and scholarship; Ukrainian history also occupied only a modest place in research and teaching in Central and Western Europe”. ^2^

So this marginalization also operated through naming – Ukrainian phenomena were often described within larger, more familiar categories: *Russia, Russian.* One of the clearest examples of this mechanism can be found in art history and museum classification. Artists connected to Ukraine by birth, education, subject matter, or cultural context have long been presented as part of *Russian art* or the *Russian avant-garde.* Recent debates around Ilya Repin, Arkhyp Kuindzhi, and Kazimir Malevich demonstrate how imperial geography can continue to shape cultural attribution long after the collapse of imperial political structures [2]. These debates do not necessarily require a simple replacement of one national label with another – they open a more productive interpretative field in which *Russian art* can no longer function as a universal container.

This broader pattern of misattribution is central to the present paper. Global comics before 2014 often reproduced preexisting habits of seeing and representing Ukraine. It would be inaccurate to claim that these patterns disappeared after 2014. Established narratives change slowly. However, they can be disrupted by major political shocks, particularly when the consequences of these transform global political and cultural perceptions. Russia’s full-scale invasion of Ukraine in 2022 created such a rupture.

The paper examines selected global comics produced before 2014 [3]. (The selection of comics analyzed in this article was drawn from the openly available dataset ^4^) The corpus is not intended to be exhaustive. The selected works include Hergé’s *Tintin in the Land of the Soviets* (1929), Spain Rodriguez’s *Nestor Makhno* (1976), and the French series *Ivan Karine* (1963–1964) [4]. These comics predate 1991 and thus belong to the period before Ukrainian independence. But they make it possible to identify earlier representational modes through which Ukrainian territories, figures, symbols, and cultural references became visible in global comics [5]. This selection allows for the examination of representation in different comic traditions: European adventure and historical-adventure comics, French popular serial comics, and American underground comics. These works differ significantly in genre, visual style, narrative structure, publication context, and target audience. Still, they are, of course, connected by their direct or indirect engagement with Ukrainian historical, spatial, or cultural material.

The paper uses the term *distorted visibility* to describe representation: Ukraine is not simply absent, but appears in ways that obscure its own name, history, and cultural specificity. This concept brings together several recurring representational mechanisms: absence, substitution, mislocation, Russification, Sovietization, and symbolic appropriation. The distinction is important because it allows us to move beyond the question of whether Ukraine appears in comics and to ask how it appears, under what names, through which visual codes, and within which geopolitical frames.

Such a question links the analysis of comics to broader debates on representation, imagined geography, and imperial knowledge. In this context, several works should be mentioned as relevant points of reference: *Representation: Cultural Representations and Signifying Practices* ^5^; Larry Wolff’s *Inventing Eastern Europe: The Map of Civilization on the Mind of the Enlightenment* ^6^; Maria Todorova’s *Imagining the Balkans* ^7^; Edward Said’s *Orientalism* ^8^; Ewa Thompson’s *Imperial Knowledge: Russian Literature and Colonialism* ^9^; Serhii Plokhy’s *The Lost Kingdom: The Quest for Empire and the Making of the Russian Nation* ^10^; Jason Dittmer’s edited volume *Comic Book Geographies.* ^11^

Methodologically, the paper combines visual, discursive, and narratological analysis. A postcolonial and decolonial perspective also informs it. The analysis focuses on four interrelated dimensions: naming, spatial framing, iconography, and narrative positioning. Naming is crucial because the presence, absence, or substitution of the word “Ukraine” determines whether a place, figure, or event becomes recognizable as Ukrainian. Spatial framing concerns how territories are mapped, located, and connected to larger geopolitical formations. Iconography refers to the visual signs through which cultural identity is suggested, displaced, or obscured. Narrative positioning concerns the function of Ukraine-related material within the story: whether it serves as historical context, political subject, exotic background, or a visual resource for another narrative.

This methodological approach allows the paper to treat comics as visual texts that participate in the production of geopolitical meaning. The following case studies examine the mechanisms mentioned earlier.

## Case Study 1: Hergé and the Homogenization of Soviet Space

Hergé’s *Tintin in the Land of the Soviets* ^12^ was the first adventure of Tintin and Snowy. It was originally serialized in *Le Petit Vingtième* from 1929 to 1930 and later published as an album. The comic is openly satirical and anti-Soviet in orientation: Tintin travels to the “land of the Soviets” as a reporter and exposes violence, propaganda, electoral manipulation, forced requisitioning, hunger, and the brutality of Soviet institutions. The Soviet Union is represented as a space of deception and coercion, where official slogans hide poverty, repression, and administrative violence.

For this paper, the comic’s importance lies in how it constructs Soviet space as a homogeneous visual and political field. The cover already establishes this logic of visual homogenization. Although the comic is set in the “land of the Soviets,” the image used to introduce this space relies on recognizably Russian-coded signs. Tintin is dressed in a stylized Russian outfit, while the well-known silhouette of onion-domed Orthodox churches dominates the background. ^12^ Before the narrative even begins, Soviet space is made legible through Russian cultural markers, while the diversity of the Soviet republics disappears from view. It mirrors the later-established representational logic by which the Soviet Union is imagined as culturally homogeneous and implicitly Russian.

This homogenization is especially significant because several episodes in the comic touch on themes connected to Ukrainian historical experience: grain requisitioning, hunger, peasants, “kulaks,” and coercive state violence in rural areas. In one sequence, Soviet officials discuss the wheat shortage and the need for grain for foreign propaganda. Their proposed solution is to organize an expedition against rich peasants and force them at gunpoint to give up their grain. ^12^ The vocabulary of wheat, hunger, requisitioning, and armed coercion evokes the politics of Soviet agricultural violence. Yet Ukraine is not named as a distinct space in this context. The scene is placed within an undifferentiated Soviet geography. The reader encounters “the Soviet Union” as a single repressive entity, rather than Ukraine, Belarus, Russia, or any other specific republic. Ukrainian historical resonance is therefore possible, but it remains unmarked. The issue is not that Hergé should have represented Ukraine; the point is that Ukrainian-related material becomes absorbed into a generalized Soviet frame.

The figure of the Cossack also complicates the scene. In one episode, a Cossack-like figure appears and hopes to receive a reward from the GPU for finding Tintin. Visually, this figure is marked through a costume associated with Cossack imagery: a long coat, cartridge holders across the chest, a fur hat, and a military posture. However, the coding is not specifically Ukrainian. It resembles the broader imperial image of the Cossack as a servant of state power and police violence, aligning more closely with Western images of the Don or Kuban Cossacks associated with the Russian Empire and the militarized Russian frontier. ^12^ The same effect is reinforced by the use of Russian words, either transcribed in Latin letters without translation, such as *nitchevo* and *bandit*, or written in Cyrillic, often with errors and also left untranslated, as in *чортъ*, *что вы ту делаете*, and *идите за мною.* These linguistic markers produce a generalized effect of Russianness and foreignness for the Western reader. Russian becomes the default linguistic marker of Soviet space, while other languages of the Soviet Union remain absent. The errors in the Cyrillic phrases further underline that language functions here as an exoticizing visual sign.

Hergé’s comic, therefore, exemplifies the first level of distorted visibility: the absence of national specificity inside the category of Soviet space.

## Case Study 2: Nestor makhno and the russian revolution frame

Spain Rodriguez’s *Nestor Makhno* is a short biographical comic devoted to the Ukrainian anarchist leader Nestor Makhno. It was first published in 1976 and later appeared in the first issue of Anarchy Comics in 1978. ^13^ The choice of Makhno by Rodriguez [6] as a protagonist was closely connected to the political and aesthetic environment of American underground comix [7], where anti-authoritarian figures, revolutionary violence, and resistance to state power were central themes. In this context, Makhno appears above all as an anarchist revolutionary: a militant horseman, a rebel leader, and an opponent of Bolshevik authority.

The political specificity of this milieu is important for understanding Rodriguez’s treatment of Makhno. The 1970s saw the development of several explicitly political lines within underground comix, including *Slow Death Funnies*, *Edu-Comics *, and *Anarchy Comics.* Unlike the ecologically oriented *Slow Death* and *Edu-Comics *, *Anarchy Comics* was defined by a more specific ideological commitment. It presented itself as “comix inspired by or based on anarchist ideas and history,” grounded in the belief that “the true terrorists are governments and corporations who hold us hostage with their armament, militaries, and intelligence activities.” ^15^ This context helps explain why Makhno was selected as a suitable subject: his biography could be framed as a story of anti-authoritarian struggle, resistance to centralized power. At the same time, this framing simplifies the historical complexity of Makhno’s political position. Although Makhno was connected to anarchist circles, including the Nabat Confederation of Anarchist Organizations, his relationship was not straightforward. Rodriguez’s comic leaves these tensions aside and presents him as an immediately legible anarchist revolutionary figure. As a result, Makhno’s Ukrainian context becomes secondary to his usefulness within Anarchy Comics’ political imagination. He appears less as a historical actor than as a recognizable icon of anti-state resistance.

The narrative situates the events within the “Russian Revolution of 1917” and identifies Ukraine as the location. In other words, the comic does not fully erase Ukraine, yet it subordinates Ukrainian space to the broader and more recognizable narrative of the Russian Revolution.

The story begins with the image of an armed horseman, a motif that would become a hallmark of later visual representations of Makhno as a mobile, militant, and charismatic figure. Yet foregrounding the horse rather than the person already emphasizes movement, force, and revolutionary action over historical specificity. The narrative is condensed into ten dynamic panels, with captions placed beneath the images. This structure gives the comic the form of a compressed illustrated biography or political legend rather than a documentary reconstruction of historical events. The absence of documentary precision is also visible in the treatment of place and visual detail. The comic does not aim to reconstruct Ukrainian landscapes, settlements, clothing, or social environments with historical accuracy. The clothing of peasants and other figures does not clearly mark the action as Ukrainian, and few visual indicators would make Ukraine legible as a distinct cultural or historical space.

The comic names several historical figures, including Wrangel, Denikin, and Trotsky. It also includes a separate frame in which Trotsky orders the elimination of anarchist elements. These references position Makhno within the conflicts of the revolutionary and civil war period. However, the comic does not substantially explore the Ukrainian context of these conflicts. It does not address the complexity of the Ukrainian Revolution, the competing projects of statehood, peasant self-organization, anti-Bolshevik resistance, or the social and regional conditions that shaped the Makhnovist movement. Ukraine functions mainly as the setting for a broader anarchist narrative.

Makhno himself is represented ambivalently. The comic presents him as brave and anti-authoritarian, but also as brutal, ruthless, and cunning. He is shown as maintaining strict discipline in his army despite the proclaimed ideals of a democratic or libertarian order. His life is framed as ending in betrayal by the Bolsheviks, exile, alcoholism, and decline. This portrayal reflects the countercultural and provocative spirit of underground comix, which often rejected sanitized heroic narratives.

In support of this paper’s argument, Rodriguez’s *Nestor Makhno* shows how Ukrainian history can be absorbed into a larger revolutionary mythology. Makhno is not Russified in the simple sense of being called Russian. Ukraine is not absent, but it is relegated to a secondary role to the more familiar and ideologically useful frame of the Russian Revolution.

Ukraine appears as a place, but not as a fully developed historical subject. The comic exemplifies substitution and Soviet/Russian revolutionary framing as mechanisms of distorted visibility: Ukrainian material is visible, but it becomes meaningful mainly through non-Ukrainian historical and ideological categories.

## Case Study 3: Cossack imagery and symbolic appropriation in ivan karine

The character of Ivan Karine was created by the Italian artist Vincenzo “Enzo” Chiomenti and first appeared in *Rodéo* no. 138 in 1963, continuing until no. 151 (1964). ^16^ Published by Lug, *Rodéo* belonged to the French *petit-format market and was associated primarily with west-oriented* adventure narratives: ranger heroes, horsemen, pursuits, armed conflict, and frontier settings. Its very title signals this orientation. These magazines typically offered compact, serialized, black-and-white adventure stories for a wide readership.

Jean-Marc Lofficier states that the creation of *Ivan Karine* was largely inspired by Jules Verne’s *Michel Strogoff* (1876) and by its popular film adaptations, including *Michel Strogoff* (1956) and *The Triumph of Michel Strogoff* (1961). This reference is important because *Michel Strogoff* belongs to a European tradition of imagining Russia and its imperial borderlands as spaces of danger, loyalty, mobility, military mission, and exotic adventure. *Ivan Karine* is constructed according to this matrix.

The character’s position within *Rodéo* also suggests a certain marginality. Ivan Karine does not appear to have become the magazine’s defining visual identity. Available evidence indicates that the series was serialized in Rodéo as a single adventure story among others, while the magazine’s covers remained primarily tied to western (American) iconography. This does not make the character insignificant, but it shows that the Ukrainian/Cossack element was incorporated into an existing adventure format and not foregrounded as a distinct cultural or historical focus.

The series ended in 1964, but it was reactivated in a new political and symbolic context after Russia’s full-scale invasion of Ukraine. In the 2022 Hexagon Comics special issue, the first episode of *Ivan Karine* ( *The Flames of Victory*) was translated into English and placed after the new *Captain Ukraine* story, where it functions as a historical and genealogical background for the modern superhero. ^17^ The paratext presents Ivan Karine as “The First Captain Ukraine” and links him to the contemporary female superhero Captain Ukraine, who is described as his descendant [8]. This retrospective framing transforms a 1960s adventure character into the origin point of a Ukrainian superhero lineage.

This later Ukrainianization, however, should not obscure the representational logic of the original series. The 1963 *Ivan Karine* was not created as a Ukrainian superhero narrative. It was shaped by French popular adventure conventions, by the western/frontier model of *Rodéo*, and by a Franco-European imagination of Russia and its imperial borderlands.

This publishing context has direct consequences for the iconographic analysis. The series presents its protagonist as a seventeenth-century Zaporozhian Cossack. Yet, the visual rendering of the Cossack figure in Western popular culture of this period rarely distinguishes among the specific material cultures of different Cossack traditions. The iconography of the Zaporozhian Cossack – wide sharovary [9], the oseledets hairstyle [10] and the strong influence of Ottoman and steppe visual culture – differs substantially from that of the Don and Kuban Cossacks [11], whose dress became increasingly standardized within the Russian imperial military system from the eighteenth century onward, incorporating the cherkesska [12] and papakha [13] under strong Caucasian influence. *Ivan Karine*’s appearance is close to that of a Don or Kuban Cossack, according to imperial Russian Cossack tradition.

In Ivan Karine, this instability is also evident in the use of naming and terminology. The protagonist is introduced as “Kotchevoy Ivan Karine”. The term “Kotchevoy” is glossed as “Captain,” but the form itself follows a Russianized transliteration rather than the Ukrainian *koshovyi.* The name Piotr also follows a Russian pattern, while the surname *Karine* does not sound Ukrainian. It may even invite a question about a possible echo of Russian literary naming – perhaps an allusion to Tolstoy’s *Anna Karenina* ….? In any case, the name does not anchor the protagonist in a recognizably Ukrainian naming tradition.

This imperial framing becomes clearer in the plot. The hetman is portrayed as a cunning, corrupt local ruler who secretly collaborates with the Tatars and puts the Cossack heroes in danger. Visually, he is drawn less as a recognizable Ukrainian Cossack leader than as an Orientalized figure, closer to the type of a pasha. Ivan, by contrast, is young, handsome, courageous, and morally transparent. The opposition between the grotesque hetman and the noble Cossack hero follows the logic of adventure fiction. Still, it also reproduces a deeper political pattern: local Ukrainian authority is marked by betrayal, while the virtuous hero proves himself through loyalty to the tsar. This structure inevitably recalls the older imperial narrative of Cossack betrayal, especially the Mazepa/Peter I paradigm, even though Mazepa is not named – the plot centers on a hetman who betrays his people and endangers Peter. Ivan Karine exposes the plot and helps save the tsar from ambush. The Cossack hero is validated not by Ukrainian autonomy but by imperial service. In this sense, Ivan Karine does not simply use Cossack imagery as picturesque decoration. It reorganizes Ukrainian-coded material into a Russian imperial adventure narrative in which the good Cossack serves “the people and the tsar.”

The episode with the *chaika* (a traditional Cossack boat used especially by the Zaporozhian Cossacks for river and sea military campaigns) [14] is revealing not because the comic should be expected to reconstruct Cossack material culture with documentary precision. *Ivan Karine* is a fictional adventure comic, and its logic depends on speed, danger, escape, and visual ingenuity rather than on historical accuracy. Yet the way the *chaika* is shown remains interesting. The comic retains the Ukrainian/Cossack term, but glosses it as “small round boats” and depicts the vessels as round, barrel-like objects that allow the heroes to escape. For readers familiar with historical Cossack chaika – long, fast boats associated with Black Sea campaigns – this creates an unintended comic effect. The comic does not seem to address readers who would recognize this discrepancy; rather, it assumes an audience for whom the foreign term and the dynamic escape scene are sufficient. This is how popular visual culture can stabilize simplified images. The same loose handling of historical geography appears in the reference to Donetsk in a seventeenth-century setting since the modern city did not exist under that name at the time.

The Chinese fireworks episode adds another layer of exoticization. A Chinese character appears in the Ukrainian village, sells fireworks, and these fireworks later help Ivan and his companions frighten the Tatars and save the Russian troops. The scene works as a highly entertaining adventure environment.

The episode follows the conventions of popular adventure fiction, which organizes Ukraine as an imperial frontier. The Cossack hero becomes readable not through Ukrainian political agency, but through loyalty to the tsar and the restoration of imperial order. Ukrainian material is present and even central to the plot, yet its meaning is reassigned through a mental map in which Eastern Europe appears as a spectacular borderland of the Russian imperial imagination.

## Closing remarks and open questions

The three case studies suggest that comics do not only reflect existing mental maps of Eastern Europe; they also help stabilize and circulate them. In *Tintin in the Land of the Soviets*, Soviet space becomes visually legible through Russian-coded signs. In *Nestor Makhno*, a Ukrainian historical figure, is made meaningful through the Russian Revolution and anarchist mythology. In Ivan Karine, Cossack imagery is placed within an adventure narrative shaped by exoticized frontier space and Russian imperial loyalty. In all three cases, Ukrainian material is visible, but its meaning is mediated by frameworks that displace Ukrainian historical and cultural specificity.

These representations are shaped by the expectations of specific genres: satire, underground political comix, and popular adventure fiction. The issue, therefore, is not whether fictional comics should be judged by the standards of historical reconstruction. But the question is how far fictional invention, simplification, and exoticization participate in the formation of broader cultural knowledge about a country, especially when the audience is unlikely to recognize historical or cultural inaccuracies.

When Ukraine repeatedly appears through Russian, Soviet, imperial, or vaguely Eastern European frames, these images contribute to long-lasting perceptual habits. These representations do not directly determine political attitudes, but they work at the level of durable cultural assumptions. If Ukraine has long been imagined as part of Russia, the Soviet Union, or an undifferentiated post-Soviet space, then later arguments about the “inseparability” of Ukrainian and Russian spaces may appear more familiar or plausible to some audiences than they should.

These observations lead to several open questions for discussion and provide directions for future research.

- How can we distinguish between the legitimate freedoms of fictional representation and the repetition of imperial mental maps?
- When does exoticization in popular culture become politically significant?
- How do comics as part of popular culture contribute to the production of knowledge about countries that are already weakly visible in global cultural circulation?
- How can pre-1991 comics that engage with Ukrainian-related material be analyzed without anachronism, while still accounting for the Russian, Soviet, and imperial frames through which this material was made visible?

## Ethics and consent

Ethical approval and consent were not required for this paper.

## Data Availability

The corpus analyzed in this study was selected from the openly available dataset Pidoprygora S. *Ukraine in Comics (1991–Present): Bibliographic Dataset.* 2026. https://doi.org/10.5281/zenodo.20626041). The dataset is available under a CC BY 4.0 licence.

## Footnotes

[1] Translation from German is mine.

[2] For example,. ^3^

[3] Japanese manga are not included in this conference paper, although representations of Ukraine in manga are relevant to the larger research project.

[4] Mainstream superhero comics, including DC and Marvel, are not considered in this paper. Although they also contain relevant representations of Ukraine, their genre conventions, serial continuity, and narrative logic require separate analysis and will be addressed at a later stage of the research.

[5] This paper forms part of a larger research project in progress. A subsequent stage of the research will examine post-1991 global comics, including Igort’s *Quaderni ucraini* (2010), Emmanuel Lepage’s *Un printemps à Tchernobyl* (2012), and Francisco Sánchez and Natacha Bustos’s *Chernobyl* (2012), in order to trace the persistence, transformation, or disruption of these earlier modes of representation before 2014.

[6] Read more about Rodriguez ^14^:.

[7] The spelling *comix* rather than *comics* was commonly used in the American underground comix movement of the 1960s–1980s to distinguish these countercultural publications from mainstream commercial comics.

[8] A separate issue containing several episodes of *Ivan Karine* was announced for publication on 1 June 2026 ^18^:.

[9] 
*Sharovary* are wide, loose trousers traditionally associated with the visual iconography of the Zaporozhian Cossacks and later with folkloric representations of Ukrainian Cossack identity.

[10] The *oseledets* is a distinctive Cossack forelock or scalp-lock, conventionally associated with Zaporozhian Cossack masculinity and military identity in Ukrainian cultural memory.

[11] For more on the Don and Kuban Cossacks in the service of the Russian Empire, including their military organization, social composition, traditions, and dress, see. ^19^

[12] A *cherkesska* is a long, fitted Caucasian coat with cartridge holders on the chest.

[13] A *papakha* is a tall wool or sheepskin hat traditionally worn in the Caucasus.

[14] For an illustration of what a Cossack *chaika* looked like, see ^20^:.
