# Supplementary material for: Visible After invasion? Ukraine’s representational absence and distortion in global comics before 2014
Source: Open Res Eur. 2026 Jul 24;6:258. [Version 1] doi: 10.12688/openreseurope.24247.1 (PMC13560625; doi:10.12688/openreseurope.24247.1)
Supplement: Peer review history

# Reviewer report: Reviewer response for version 1

**DOI:** 10.21956/openreseurope.26256.r78084

**Published:** 2026-08-28

**Authors:** Silke Pasewalck

**Affiliations:** 1 The Bundesinstitut für Kultur und Geschichte der Deutschen im östlichen Europa (BKGE), Oldenburg, Germany

The paper's research question is how Ukraine has been shaped in globald comics published before 2014. Regarding this topic the article's main title “Visible after invasion?” is kind of misleading as it sets the focus on the present – although the article examines comics published before Russia's annexation of Crimea.

 Three case studies from different comic traditions have been chosen and examined: *Tintin in the Land of the Soviets* (1929) by Hergé, *Nestor Makhno* (1976) by Spain Rodriguez, and *Ivan Karine* (1963–1964) by Enzo Chiomenti. The paper analyses four dimensions of representation: naming, spatial framing, iconography, and narrative positioning. It introduces the concept of distorted visibility to describe representational mechanisms such as absence, substitution, Russification, Sovietization, symbolic appropriation, and mislocation.

 .

 In terms of methodology the author combines visual, discursive and narratological analysis, which is quite convincing. Specific comic analytical methods are nevertheless missing.

 The conclusions drawn by the data and analysis are adequate and convincing. One might only ask if these results were not to be expected bearing the Soviet and Russian narratives in popular culture in general in mind.

Is the case presented with sufficient detail to be useful for teaching or other practitioners?

Yes

Is the work clearly and accurately presented and does it cite the current literature?

Yes

If applicable, is the statistical analysis and its interpretation appropriate?

Yes

Are all the source data underlying the results available to ensure full access and reuse by other researchers?

Yes

Are the conclusions drawn adequately supported by the data and analysis?

Yes

Is the background of the case’s history and progression described in sufficient detail?

Yes

Reviewer Expertise:

Literature studies and comic studies

I confirm that I have read this submission and believe that I have an appropriate level of expertise to confirm that it is of an acceptable scientific standard.

# Reviewer report: Reviewer response for version 1

**DOI:** 10.21956/openreseurope.26256.r78077

**Published:** 2026-08-19

**Authors:** Adam Twycross

**Affiliations:** 1 Anglia Ruskin University, Cambridge, UK

This paper argues that depictions of Ukraine in global comics prior to 2014 were characterised by an inauthenticity which derived from a tendency to portray the country through mediating frameworks that replaced Ukrainian specificity with more generalised Russian, Soviet or Eastern European representational modes. Three case studies are chosen to illustrate this pattern, with examples spanning a nearly fifty-year timeframe and emerging from distinct geographic and temporal contexts that include European adventure comics, French popular serial comics and American underground comics. Using the term *distorted visibility* to identify the (mis)representation of Ukraine identifiable within these texts, the author identifies four principle mechanisms at work: naming, spatial framing, iconography, and narrative positioning. Methodologically, the paper uses a combination of visual, discursive and narratological analysis and features an influence from numerous pertinent scholarly texts from the last two decades.

 The paper appears as part of a larger body of work and, perhaps as a result of this, feels at times as if further exploration and examination of key ideas would be appropriate. For example, on the third page the author notes that existing scholarly attention has been paid to the way in which Ukrainian art and artists have often been absorbed into the classificatory systems of *Russian Art* or the *Russian Avant Garde*. A rejection of such imagined or imperial geographies is posited to require more than a simple replacing of one national label with another; what alternative paradigms might be possible are not explored in depth, however, and thus a very interesting avenue is not exploited to the full.

 Similarly, on the seventh page, the discussion of the exoticisation present in *Ivan Karine* includes a short paragraph covering the use of Chinese fireworks as a narrative motif; the discussion is very brief, however, and would thus benefit from further extension or, perhaps, from absorption into the body of another paragraph.

 One other area for possible exploration within the wider project concerns the possibility that contemporary expectations that Ukrainian cultural practice be authentically portrayed might conflict with the historic intention embodied by the texts under review. The discussion of *Tintin in the Land of the Soviets*, for example, notes that the depiction of the Soviet Union is homogenised and portrayed as implicitly Russian, with the result that Ukraine is not named as a distinct space. The text is, however, clearly engaged in a critique of the Soviet Union and the agricultural violence enacted within it, and it is not immediately clear how identifying Ukraine, or any other specific Soviet state, as the backdrop to the depicted acts would strengthen what is clearly intended to be an attack on Soviet policy writ large.

 In the main, the above observations are intended to offer some thoughts on how the presented research might usefully be extended or clarified within the wider ongoing project. Taken as a whole, the paper represents a timely and well-argued overview of some of the ways in which the cultural specificity of Ukraine has been inauthentically represented in historic comics from wide geographic and temporal contexts. The paper extends existing scholarly work on imagined and imperial geographies and clearly identifies areas for further and deeper research.

Is the case presented with sufficient detail to be useful for teaching or other practitioners?

Yes

Is the work clearly and accurately presented and does it cite the current literature?

Yes

If applicable, is the statistical analysis and its interpretation appropriate?

Not applicable

Are all the source data underlying the results available to ensure full access and reuse by other researchers?

No source data required

Are the conclusions drawn adequately supported by the data and analysis?

Yes

Is the background of the case’s history and progression described in sufficient detail?

Yes

Reviewer Expertise:

Comics Studies, Historical Practice, Newspapers, Adult Comics

I confirm that I have read this submission and believe that I have an appropriate level of expertise to confirm that it is of an acceptable scientific standard.

# Reviewer report: Reviewer response for version 1

**DOI:** 10.21956/openreseurope.26256.r78080

**Published:** 2026-08-12

**Authors:** Nina Mickwitz

**Affiliations:** 1 University of the Arts London, London, UK

Representing a strand of a larger project, this research article examines three 20th century comics as instances of how, until relatively recently, Ukraine remained largely misrecognized in Western political and scholarly discourse and cultural imaginaries.  The author engages the concept of *distorted visibility*, dynamically connected to epistemic misrecognition, and uses case study analysis to illustrate specific mechanisms such as ‘naming, spatial framing, iconography and narrative positioning’ (Pidoprygora 2026, p. 4 of 8) to productive effect. This is an informative and engaging piece of writing, and the overall argumentation is well-structured and persuasive.

 On occasion, some further background information could be helpful, especially for readers whose knowledge of Ukrainian culture and history has been shaped by the very mechanisms identified in the analysis. For instance, it would be useful to give a little more introductory information about Cossacks, eg the historical background of this warrior grouping and the way Cossack imagery is important to, and embedded in, Ukrainian national iconography. A case in point for the broader argument, this is a figure that for many outside Ukraine, has simply been (mis)understood as a signifier of 'Russianness'). For this reason, a more detailed explanation of ‘the older imperial narrative of Cossack betrayal’ and the Mazepa/Peter I paradigm’ (p. 7/8), including the identity of Mazepa, would help readers with limited grasp of these historical events.

 Some brief mention of the political backdrop for *Tintin in the Land of the Soviets* would likewise seem appropriate and offer a useful reminder that this story emerges from a specific historical milieu. Pages 17-19 in Apostolidès, J.M., 2010. *The Metamorphoses of Tintin: Or Tintin for Adults*. Stanford University Press. (accessible in Google Books) set out the background for its anti-communist stance.

 Case Study 2, Spain Rodriguez’ (1976) *Nestor Makhno* is an interesting illustration of how historical narratives are shaped by the positionality of their telling and of narrators - an early historiographic account of this was Hayden White.

 White, Hayden (1984) 'The Question of Narrative in Contemporary Historical Theory.'

*History and Theory, Vol. 23, No. 1* *https://www.jstor.org/stable/2504969*

 White's argument was about history as a scholarly discipline.  This idea seem might less controversial, not only forty years after it was first published, but also in the context of popular culture. Nevertheless, this work comes to mind as relevant to the discussion.

 To sum up, this article offers valuable, original and overdue insights into the mechanisms through which Ukraine has been obscured and rendered invisible through transnational cultural representational schema. In its attention to comics, the analysis effectively highlights the links between epistemology, geo-political power-dynamics and mechanisms through which ‘durable cultural assumptions’ (p. 8/8) circulate.

 Minor formatting errors: Footnote 2 is incomplete. Footnote 5 appears misplaced. This content seems important enough to be moved, not only into the main text but possibly to the start of the paragraph currently beginning: ‘The paper examines selected global comics produced before 2014.’ This would bridge the shift from the initial context to the paper itself. Footnote 6 also incomplete. Roger Sabin writes about Rodriguez in *Adult Comics* (1993, 2010), possibly also in the *Comics, Comix and Graphic Novels *(2001). The first paragraph of Case Study 3 includes extended italics that seem out of place (p. 6/8). In the heading for Case Study 2, capitalise ‘Russian’ and ‘Makhno’ for consistency.

 I am not sure if the references are formatted according to a given system, in which case the following is irrelevant. But, if this is not the case: for clarity and to more clearly separate the numbered footnotes from references, consider removing all numbers from the references and instead list them alphabetically by author surname at the end. Add publication dates to in-text mentions: Kappeler (2015) etc. When referencing sources in footnotes 11 and 14, you would then give author names and dates instead of numbers, eg (Bloch 2013) and (Cherkas 2013). If the latter is a web source, please add the URL: https://www.istpravda.com.ua/articles/2013/10/10/137416/ and [date accessed], to the reference list, as it would make this reference more user friendly.

Is the case presented with sufficient detail to be useful for teaching or other practitioners?

Yes

Is the work clearly and accurately presented and does it cite the current literature?

Yes

If applicable, is the statistical analysis and its interpretation appropriate?

Yes

Are all the source data underlying the results available to ensure full access and reuse by other researchers?

No source data required

Are the conclusions drawn adequately supported by the data and analysis?

Yes

Is the background of the case’s history and progression described in sufficient detail?

Yes

Reviewer Expertise:

Comics Studies, Documentary Comics, Cultural Theory and Popular Culture

I confirm that I have read this submission and believe that I have an appropriate level of expertise to confirm that it is of an acceptable scientific standard.
